# Distribution of *Mycobacterium ulcerans* in Buruli Ulcer Endemic and Non-Endemic Aquatic Sites in Ghana

**DOI:** 10.1371/journal.pntd.0000205

**Published:** 2008-03-26

**Authors:** Heather R. Williamson, Mark E. Benbow, Khoa D. Nguyen, Dia C. Beachboard, Ryan K. Kimbirauskas, Mollie D. McIntosh, Charles Quaye, Edwin O. Ampadu, Daniel Boakye, Richard W. Merritt, Pamela L. C. Small

**Affiliations:** 1 Department of Microbiology, University of Tennessee, Knoxville, Tennessee, United States of America; 2 Department of Entomology, Michigan State University, East Lansing, Michigan, United States of America; 3 Noguchi Memorial Institute of Medical Research, Legon, Accra, Ghana; 4 National Buruli ulcer Control Programme, Disease Control Unit - GHS, Accra, Ghana; Institut Pasteur, France

## Abstract

*Mycobacterium ulcerans*, the causative agent of Buruli ulcer, is an emerging environmental bacterium in Australia and West Africa. The primary risk factor associated with Buruli ulcer is proximity to slow moving water. Environmental constraints for disease are shown by the absence of infection in arid regions of infected countries. A particularly mysterious aspect of Buruli ulcer is the fact that endemic and non-endemic villages may be only a few kilometers apart within the same watershed. Recent studies suggest that aquatic invertebrate species may serve as reservoirs for *M. ulcerans*, although transmission pathways remain unknown. Systematic studies of the distribution of *M. ulcerans* in the environment using standard ecological methods have not been reported. Here we present results from the first study based on random sampling of endemic and non-endemic sites. In this study PCR-based methods, along with biofilm collections, have been used to map the presence of *M. ulcerans* within 26 aquatic sites in Ghana. Results suggest that *M. ulcerans* is present in both endemic and non-endemic sites and that variable number tandem repeat (VNTR) profiling can be used to follow chains of transmission from the environment to humans. Our results suggesting that the distribution of *M. ulcerans* is far broader than the distribution of human disease is characteristic of environmental pathogens. These findings imply that focal demography, along with patterns of human water contact, may play a major role in transmission of Buruli ulcer.

## Author Summary

Buruli ulcer is an ulcerative skin disease caused by *Mycobacterium ulcerans*. Though usually not fatal, ulceration can cover up to 15% of the body, with treatment being costly and sometimes painful. Primary risk for Buruli ulcer in Africa is exposure to stagnant water, but the route of transmission is unknown. Detection of *M. ulcerans* in aquatic insects in endemic sites suggests the presence of aquatic reservoirs. This article reports results from the first investigation into the ecology of *M. ulcerans* based on random sampling of both endemic and non-endemic aquatic sites. Development of a method for discriminating *M. ulcerans* from closely related mycobacterial pathogens made it possible to determine the distribution of *M. ulcerans* in aquatic environments. This article demonstrates the presence of *M. ulcerans* DNA in both endemic and non-endemic sites within aquatic insects, water filtrate, and glass-slide biofilm communities. This article provides data suggesting that *M. ulcerans* is more broadly distributed than the human disease it causes. This study provides an initial step for future work on whether certain *M. ulcerans* strains are particularly successful human pathogens and suggests that research on specific human water contact factors may provide insight into the transmission of *M. ulcerans.*

## Introduction

**Figure 1:**
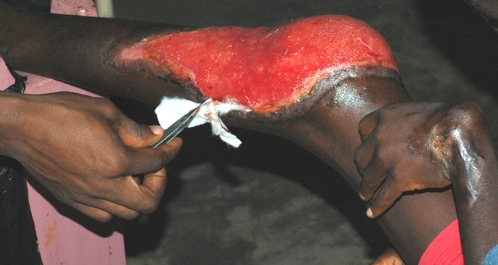
Buruli ulcer on the left limb following surgical debridement. Also shown is joint contracture of the left wrist from scarring caused by Buruli ulcer.

*Mycobacterium ulcerans* is the cause of Buruli ulcer, a severe necrotizing skin infection (Figure 1). Although Buruli ulcer is globally distributed, it is an emerging infection primarily in Australia and West Africa [1]. The disease begins as a painless nodule or papule that, if left untreated, can lead to extensive ulceration that could cover 15% of the body [2]. Though the disease is not usually fatal, Buruli ulcer can lead to profound morbidity, especially within rural areas of West Africa where treatment options are limited. Though sex and age are not seemingly risk factors, women and children between the ages 5 and 15 are most often infected. Incidence of Buruli ulcer has increased over the last several years. For instance, in Ghana, the number of new cases reported has been 685 in 2003, 1021 in 2004, 1097 in 2005, and 1010 in 2006. True incidence data, however is difficult to determine due to poor surveillance measures and case confirmation.

The major virulence determinant for *M. ulcerans* is a plasmid-encoded toxic macrolide, mycolactone [2],[3]. Acquisition of the mycolactone plasmid is thought to have been a pivotal event in the evolution of *M. ulcerans* from an *M. marinum-*like ancestor [4]. Like *M. marinum*, *M. ulcerans* is an environmental pathogen. Although the exact mode of transmission for *M. ulcerans* remains unknown, person to person transmission is extremely rare and a large body of epidemiological data supports the hypothesis that infection results from exposure to aquatic environments [5],[6],[7]. Lack of direct person-to-person transmission is a characteristic *M. ulcerans* shares with other environmental pathogens such as *Francisella tularensis* and *Borrelia burgdorferi*. Environmental pathogens are maintained in the environment in the absence of humans. The distribution of such pathogens is far broader than the cases of human disease. For example the life cycle of *Borrelia burgdorferi*, the causative agent of Lyme disease involves several species of *Ixodes* ticks and a number of mammalian vectors. Human infections only occur through exposure to ticks; and humans are a dead end for infection. In some areas of the Western U.S., *Borrelia burgdorferi* is vectored by an *Ixodes* species which feeds primarily on lizards and rarely bites humans. In these areas, despite the abundance of *Borrelia burgdorferi* in the environment, human Lyme disease is extremely rare [8],[9].

A major advance in deciphering the ecology of *M. ulcerans* resulted from the identification of an insertion sequence, IS*2404*, which is present in over two-hundred copies in *M. ulcerans* [10],[11]. Early work showed that IS*2404* was present in *M. ulcerans*, but absent in the closely related mycobacterial species *M. marinum*, and over 40 other mycobacterial species suggesting that the insertion sequence was specific for *M. ulcerans* [11]. In the past 15 years a large number of environmental samples collected from Buruli ulcer endemic regions in Australia and West Africa have been analyzed using IS*2404* PCR. In Australia, IS*2404* has been detected in water as well as from detritus collected from water bodies and, most recently, from trapped mosquitoes [12],[13],[14]. No acid-fast bacilli were reported and attempted cultures were negative. In 1999, Portaels *et al* reported detection of IS*2404* positive PCR from two groups of predaceous aquatic insects, Naucoridae and Belostomatidae [15]. IS*2404* PCR positive results have also been obtained from Naucoridae and Belostomatidae collected in Ghana, Cote d'Ivoire and Benin [14],[16],[17]. In 2004, Marsollier *et al* obtained IS*2404* positive PCR results from 5/80 Naucoridae collected in Cote d'Ivoire and, more importantly, successfully cultured an IS*2404* positive mycobacteria from two of these. Although one of these isolates produced an ulcer upon injection into mice, both isolates were lost before they could be fully characterized. More recently, an exciting discovery was the culture and complete characterization of *M. ulcerans* from a Gerridae, or water strider (in press). Gerridae, like Naucoridae and Belostomatidae are predacious aquatic insects in the Order Hemiptera. Unlike Naucoridae and Belostomatidae, Gerridae are unable to bite humans. However, it is likely that Gerridae along with other invertebrates share a food web with *M. ulcerans. M. ulcerans* has also been shown to form biofilms on aquatic plants [18]. A culture of an IS*2404* mycobacteria was obtained from an IS*2404* PCR positive plant (Family: Scrophulariaceae) collected from the Lobo River in Cote d' Ivoire and it has been suggested that snails may be transiently infected by feeding on this vegetation [18]. Contamination of this sample with *M. szulgai* prevented isolation of *M. ulcerans.* IS*2404* positive samples include detritus, snails, and fish [13],[17]. Taken together these results suggest that the ecology of *M. ulcerans* is complex and includes participation in a food web comprised of many different taxa and feeding groups.

Considerable speculation concerning the possibility of an insect vector for Buruli ulcer has followed from elegant studies in which laboratory infections of naucorids collected in France with *M. ulcerans* could be transmitted to mice from the bite of the infected insect [15]. However, these results have been interpreted with caution [19]. Most attempts to culture the organism from environmental sources have not been successful despite the fact that it is readily cultured from human tissues. None of the IS*2404* PCR-positive insects identified in West Africa are blood feeders, making it unlikely that they could play a major role in transmission. Further, IS*2404* has been found in several aquatic mycobacterial pathogens closely related to *M. ulcerans* such as *M. liflandii* [20], *M. pseudoshottsii* [21], and a newly discovered clade of *M. marinum* [22] isolated from frogs and fish. Finally, environmental sampling has not been conducted in a systematic way and results from samples collected in non-endemic regions have not been conducted with the exception of one study in which an unspecified number of unidentified plants were collected from Cote d' Ivoire [18].

A problem inherent in the identification of pathogens in the environment is the difficulty of distinguishing the target species within a complex and largely unknown population of background microbial flora. Although it is impossible to have complete confidence that any PCR primer set targeting a specific gene sequence is 100% specific in this context, the use of multiple PCR targets is likely to increase specificity. The completion of the *M. marinum* and *M. ulcerans* genome sequencing projects has led to the identification of variable number tandem repeat (VNTR) sequences which have been very useful in detecting heterogeneity among *M. ulcerans* [23],[24],[25]. Results from these studies suggested that it might be possible to trace transmission pathways by matching VNTR profiles from environmental samples with those from *M. ulcerans* cultures obtained from patients in the same geographic area.

In this report we present data from a systematic collection of over 1400 environmental samples collected from both endemic and non-endemic regions of Ghana as part of a larger study aimed at defining the ecology of *M. ulcerans.* Using a tiered PCR based detection method we have mapped the distribution of *M. ulcerans* within 26 aquatic sites in Ghana. Samples analyzed include vertebrates, invertebrates, suspended solids from water filtrate, soil, and biofilms collected on glass slides. Preliminary evidence for *M. ulcerans* in environmental samples was obtained from PCR detection of the insertion sequence IS*2404* [10] along with PCR detection of the enoyl reductase (ER) domain of the mycolactone toxin [26]. Variable number tandem repeat (VNTR) analysis of ER-PCR positive samples allowed the discrimination of *M. ulcerans* from other mycolactone producing mycobacteria and also made it possible to match VNTR profiles from environmental samples with VNTR profiles obtained from patient isolates from the same region. Although *M. ulcerans* has been detected in many Buruli ulcer endemic areas of West Africa using IS*2404*-PCR [17], this is the first study in which both endemic and non-endemic sites have been randomly and systematically sampled.

The major finding from this work is that *M. ulcerans* and other mycolactone producing mycobacteria (MPM) are widely distributed in water bodies in endemic and non-endemic villages within the Ashanti and Greater Accra regions of Ghana. This is entirely consistent with *M. ulcerans's* position as an environmental pathogen. Although the human host may play a role in the dispersion of an environmental pathogen, the pathogen does not depend on the human host for dispersion. Thus the distribution of an environmental pathogen is always much broader than the distribution of disease. Further, these studies suggest that the presence of *M. ulcerans* in the environment, while necessary, is not sufficient for Buruli ulcer.

## Materials and Methods

### Bacterial strains

Strains used in this study are listed in Table 1. *M. ulcerans* strains were grown at 32°C for 4–6 weeks on M7H10 agar media. *M. liflandii* was grown at 32°C in 5% CO_2_ for 6 weeks on Bordet-gengou media. *M. pseudoshotsii*, and *M. marinum* DL strains were grown at 25°C for 6 weeks on Bordet-gengou media.

**Table 1:** Ghanaian isolates of *M. ulcerans* and other mycolactone producing mycobacteria.

Species	Strain	Source	Reference
*M. ulcerans*	1054	Human Ghanaian isolate (Central Region)	This Work
*M. ulcerans*	1055	Human Ghanaian isolate (Central Region)	This Work
*M. ulcerans*	1057	Human Ghanaian isolate (Central Region)	This Work
*M. ulcerans*	1059	Human Ghanaian isolate (Central Region)	This Work
*M. ulcerans*	1063	Human Ghanaian isolate (Ashanti Region)	This Work
*M. ulcerans*	Agy99	Human Ghanaian isolate (Greater Accra Region)	[36]
*M. marinum*	DL150991	Sea bass *Dicentrarchus labrax* (Atlit-Mediterranean Sea, Israel)	[22]
*M. marinum*	DL240490	Sea bass *Dicentrarchus labrax* (Red Sea, Israel)	[22]
*M. marinum*	DL045	Sea bass *Dicentrarchus labrax* (Mediterranean Sea, Greece)	[22]
*M. marinum*	DL300/04	Sea bass *Dicentrarchus labrax* (Mediterranean Sea, Italy)	[26]
*M. marinum*	DL180892	Sea bass *Dicentrarchus labrax* (Ein Yahav, Israel)	[22]
*M. marinum*	SA200695	Sea bream *Sparus aurata* (Red Sea, Israel)	[22]
*M. marinum*	CC240299	Koi *Cyprinus carpio* (Ma'agan Michael, Israel)	[22]
*M. marinum*	BB170200	Silver perch *Bidyanus bidyanus* (Dor-Ma'agan Michael, Israel)	[22]
*M. marinum*	CF030494	Butterflyfish *Chaetodon fasciatus* (Red Sea, Israel)	[22]
*M. marinum*	SR030597	Rabbitfish *Siganus rivulatus* (Red Sea, Israel)	[22]
*M. marinum*	Hybrid270995	Red seabream *Pagrus major* (f) x *Sparus aurata* (m) (Red Sea, Israel)	[22]
*M. Pseudoshotsii*	L15	Sea bass *Morone saxatilis*	[21]
*M. Pseudoshotsii*	L58	Sea bass *Morone saxatilis*	[21]
*M. liflandii*	Xt128F	African clawed frog *Xenopus tropicalis*	[31]
*M. liflandii*	X15	African clawed frog *Xenopus laevis*	[31]
*M. liflandii*	1138	African clawed frog *Xenopus laevis*, University of Massachusetts	This Work

### Aquatic environmental sample collection

#### a. Macroinvertebrate/vertebrate sampling

Sites were chosen based primarily upon human Buruli ulcer case endemicity defined at the district or community level from data obtained from the Ghana Ministry of Health and secondarily on logistical feasibility from 2004–2006. Water sites included small rivers, natural wetlands, as well as man-made ponds. Sites varied with respect to depth as well as exposure to sunlight and shade. Sampling was conducted between 10:00 am and 2:00 pm for all sites and sampling methods were the same for every site. Typically, benthic sampling took place at 1.5 m or less. A total of 1356 invertebrate and vertebrate samples were collected from the study sites (Table 2 and Table S1) by first establishing two transects parallel to the shoreline of each water body using measuring tape. Each transect was established through the dominant vegetation type present in the water body adjacent to the entrance point for human use. Within each transect, three randomly chosen 1 m^2^ quadrats were established where standardized samples of macroinvertebrates and vertebrates (e.g., tadpoles and small fish) were collected with a D-frame net using three 1 m sweeps that encompassed the entire water column of the quadrat. This standardized collection method allowed unbiased comparisons between sites. In addition, 30, 1 m sweep samples were also taken from representative habitats of each water-body. These samples were sieved through a 500-micron sieve and placed into a bucket for sorting out large pieces of organic matter. The remaining sample was preserved in 100% ethanol and kept in a cooler during transport while in the field and to Michigan State University, East Lansing, Michigan for taxa identification. Additional belostomatids and naucorids were selectively collected by sweep sampling as above until an appropriate amount of each was obtained.

**Table 2:** Presumptive identification of *M. ulcerans* in aquatic vertebrate and invertebrate samples in Ghana 2004–2006.

Order	Family	ER positive Samples^1^
Anura order		4/31
Araneae		8/27
Coleoptera	Noteridae	1/38
Coleoptera	Hydrophilidae	5/55
Coleoptera	Dytiscidae	3/44
Coleoptera	Elmidae	2/5
Coleoptera	Scirtidae	1/10
Coleoptera	Hydraenidae	2/14
Diptera	Chironomidae	4/57
Diptera	Culicidae	2/30
Diptera	Psychodidae	1/1
Ephemeroptera	Protoneuridae	4/24
Ephemeroptera	Caenidae	3/28
Ephemeroptera	Baetidae	2/44
Gastropoda	Physidae	1/9
Hemiptera	Notonectidae	3/37
Hemiptera	Belostomatidae	5/41
Hemiptera	Nepidae	4/21
Hemiptera	Naucoridae	3/18
Hirudinea		3/25
Lepidoptera	Crambidae	2/3
Odonata	Libellulidae	4/26
Oligochaeta		2/24
Osteichthyes		1/24
Ostracoda		2/14
Basommatophora	Planorbidae (Bulininae)	2/29
Bivalva	Sphaeriidae	1/1
Bivalva	Corbiculidae	1/1
Diptera	Sciomycidae	1/3
Diptera	Syrphidae	1/2

1 Large samples were tested individually. Smaller samples with numbers of individuals of three or above were pooled in sets of 3–15. Denominator represents total number of pooled or individual samples collected from the specific taxon.

#### b. Collections of water filtrate

At each site, ten, 100–200 mL water samples were collected from mid-water column and passed through a 1.6 micron fiberglass filter (Whatman Inc). This filtrate was then passed through a 0.2 micron nitrocellulose filter (Whatman Inc). Three additional filtrate samples were collected from each site in which water was collected mid-water column and filtered directly through a 0.2 micron nitrocellulose filter. Filters were sealed in foil packets and kept in a cooler for transport in the field and to the University of Tennessee, Knoxville, Tennessee.

#### c. Soil sampling

Three samples of soil (approximately 5 grams) were taken from the floor of each water body. Two additional soil samples were taken from the riparian zone; one at the water edge and one 5 m from the water edge.

#### d. Biofilm collection

Two sites were chosen for biofilm collection. Adigon was classified as a non-endemic site based upon incidence data from the Ghana Ministry of Health. Amasaman was classified as an endemic site for Buruli ulcer. Sixteen glass microscope slides were affixed to six PVC pipes using paper tape. The pipes with attached slides were submerged and anchored into the water body floor. Slides were collected at 3, 6 and 14 weeks and placed into individual 50 mL falcon tubes (BD Biosciences). A sub-sample was taken from one side of each slide for DNA extraction and PCR (methods described below). The remaining side of the slide was stained for acid-fast bacteria using the Kinyoun's staining according to the manufacturer's instructions (Difco).

### DNA extraction

DNA was extracted using a protocol adapted from Lamour and Finley [27]. Small invertebrates collected in Ghana were sampled in pools of groups of 3–15, whereas vertebrates and larger invertebrates were tested individually. Invertebrate samples were also collected from Tennessee. These samples were used as negative controls for PCR analysis. DNA was also extracted from *M. ulcerans Agy*99, *M. marinum* 1218, or water for use as positive and negative controls. Samples were vortexed in 400 µL lysis solution (100 mM Tris (pH8.0), 50 mM EDTA, 500 mM NaCl, 1.33% SDS and 0.2 mg/mL RNase A) and one gram 1.0 mm glass beads (Sigma-Aldrich), then centrifuged. One hundred-fifty microliters of 5 M potassium acetate was added, and each sample was incubated at −20°C overnight. Following centrifugation, supernatants were transferred to new tubes containing 0.66 M guanidine hydrochloride and 63.3% ethanol solution. The samples were then added to a MOBIO spin filter (MOBIO) in a 2 mL microcentrifuge tube (MOBIO). The flow-through was discarded and the filter was washed with 500 µL wash solution (10 mM Tris [pH 8], 1 mM EDTA, 50 mM NaCl, 67% ethanol), then further washed by the addition of 500 µL of 95% ethanol. The spin filter was dried by centrifugation, and then transferred to a new 2.0 mL microcentrifuge tube. Two-hundred microliters of elution solution (10 mM Tris [pH 8]) were added to the spin filters which were allowed to incubate at room temperature for 15 minutes. Following this, the DNA was eluted. The DNA was stored at −20°C until further use. DNA was subjected to amplification of IS*2404*, the enoyl reductase domain, and various variable number tandem repeat (VNTR) loci. Those yielding no amplification of the ER domain were diluted ten-fold twice for determination of inhibition of PCR.

### Assay for sensitivity of IS*2404* and ER

Dilutions were made of *M. ulcerans* Agy99 by first placing a loopful of cells into 1 mL of 1% SDS. Aggregates were broken by passing the suspension through a 25 gauge needle 10 times. One hundred microliters were then transferred into a new tube containing 900 µL 1% SDS, and 10-fold dilutions were made. Ten microliters of each suspension was plated in triplicate onto M7H10 plates and allowed to incubate at 32°C for 4–6 weeks at which time colony forming units were counted.

### Preparation of spiked samples for ER and VNTR analysis

In order to determine sensitivity of primer sets targeting ER and VNTR loci within environmental samples, belostomatids were spiked with serial dilutions of *M. ulcerans* DNA. Eight sacrificed belostomatid samples, each with a wet weight of 160 mg, were placed in separate vials. These vials were spiked with dilutions of *M. ulcerans* DNA (prepared as above with the exception that *M. ulcerans* 1615 was used for this study) with predicted concentrations ranging from 10^5^ CFU to .01 CFU. DNA was extracted as described.

### Primers, PCR conditions, and sequencing

Primers used for this study are listed in Table 3. A 719 basepair fragment of the enoyl reductase (ER) domain, found on one polyketide synthase gene partially responsible for toxin production, was amplified for samples as well as for *M. ulcerans Agy*99 and 1615, *M. marinum* 1218, and water (as positive and negative controls) using a 50 µL reaction mixture containing 1 µL each of forward and reverse primer (1.0 µM), 10 µL 5× Go Taq reaction buffer (Promega), 1 µL 10 mM PCR nucleotide mix (Promega), 31.7 µL ddH_2_O, 1.6 units of Go Taq polymerase enzyme (Promega), and 5 µL DNA template. An average concentration of 10 ng/µL mycobacterial DNA was used for positive and negative controls. Cycling conditions began with an initial denaturation at 94°C for 5 minutes, 35 cycles of 94°C for 1 minute, 58°C for 45 seconds, 72°C for 1 minute, and a final extension of 72°C for 10 minutes. Primers and PCR conditions for amplification of VNTR MIRU 1 and 9, and loci 4, 5, 6, 8, 14, 15, 18, 19, 33, and ST1 as well as for IS*2404* were as previously described [23],[24],[25],[11].

**Table 3:** Primers used for identification of *M. ulcerans* in the environment.

Primer Name	Forward Primer Sequence (5′ to 3′)	Reverse Primer Sequence (5′ to 3′)	Reference
ER	GAGATCGGTCCCGACGTCTAC	GGCTTGACTCATGTCACGTAAG	[31]
IS*2404*	AGCGACCCCAGTGGATTGGT	CGGTGATCAAGCGTTCACGA	[11]
MIRU1	GCTGGTTCATGCGTGGAAG	GCCCTCGGGAATGTGGTT	[24]
Locus4	GCCTTGCTTACCGTCGTGCCAA	CGAGCCAAGTTGGACCGTCAACACAT	[25]
Locus6	GACCGTCATGTCGTTCGATCCTAGT	GACATCGAAGAGGTGTGCCGTCT	[25]
Locus8	CGGATGACGTCGGAACTCTGA	GGACGCGGTAGCACGTTTTGT	[25]
MIRU9	GCCGAAGCCTTGTTGGACG	GGTTTCCCGCAGCATCTCG	[24]
Locus14	CCTTGTATCCGAGTTTCAGTT	GTCGACCAGATATGAGCAAT	[25]
Locus15	GCCACCGGTCAGGTCAGGTT	TCACCAACTACGACGGCGTTC	[25]
Locus18	CCCGGAATTGCTGATCGTGTA	GGTGCGCAGACTGGGTCTTA	[25]
Locus19	CCGACGGATGAATCTGTAGGT	TGGCGACGATCGAGTCTC	[25]
ST1	CTGAGGGGATTTCACGACCAG	CTGAGGGGATTTCACGACCAG	[23]

The amplified DNA was subjected to gel electrophoresis using a 1.5–3.0% agarose gel and band sizes were compared using a 1 Kb DNA ladder (Invitrogen).

PCR products from all positive samples were cloned into the pCR2.1 Topo vector (Invitrogen) and sequenced using an ABI 3100 automated genetic analyzer (Applied Biosystems).

## Results

### Comparison of a mycolactone-based enoyl reductase (ER) PCR target to an IS*2404* PCR target for detection of *M. ulcerans*

IS*2404* PCR has been widely used for detection of *M. ulcerans* in the environment and patients because of the high copy number of the IS element (213) within the *M. ulcerans* genome [28] However, evidence from the *M. ulcerans* genome as well as results from restriction fragment length polymorphisms of IS*2404* suggests considerable heterogeneity between copies, as well as the presence of incomplete copies which could lead to production of multiple products [29]. For this reason we developed a PCR method based on amplification of the ER domain of *mlsA* which encodes a polyketide synthase that produces the mycolactone core, and compared the sensitivity of ER PCR and IS*2404* PCR using environmental samples, as well as *M. ulcerans* cultures.

In this study 319 invertebrate and vertebrate samples were analyzed using IS*2404* PCR and the PCR products were sequenced. A PCR product of appropriate size was obtained from eight invertebrate samples. However, DNA sequencing showed that only four of the samples contained IS*2404* DNA. Although adjustment of PCR parameters improved specificity somewhat, many non-specific products were still amplified. ER PCR of the initial eight IS*2404* positive samples yielded four ER positive samples. DNA sequence results confirmed that all four ER positive samples contained ER sequence. Further analysis of DNA from 71 ER positive samples showed that ER DNA was the product in every case.

**Figure 2:**
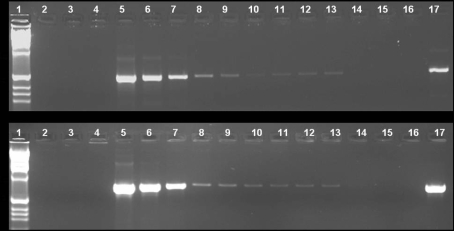
Comparison of IS*2404* and ER PCR for detection of *M. ulcerans.* Lanes represent serial dilutions of *M. ulcerans* 1615 from 10^7^ to 10^−4^ CFU detected using probes for IS*2404* (A) or ER (B). Lanes are labeled 1: 1KB ladder; 2: water blank for DNA extraction; 3: water blank for PCR; 4: *M. marinum* 1218; 5: *M. ulcerans* 1615 10^7^ CFU; 6: *M. ulcerans* 1615 10^6^ CFU; 7: *M. ulcerans* 1615 10^5^ CFU; 8: *M. ulcerans* 1615 10^4^ CFU; 9: *M. ulcerans* 1615 10^3^ CFU; 10: *M. ulcerans* 1615 10^2^ CFU; 11: *M. ulcerans* 1615 10^1^ CFU; 12: *M. ulcerans* 1615 1 CFU; 13: *M. ulcerans* 1615 10^−1^ CFU; 14: *M. ulcerans* 1615 10^−2^ CFU; 15: *M. ulcerans* 1615 10^−3^ CFU; 16: *M. ulcerans* 1615 10^−4^ CFU; 17: *M. ulcerans* 1615 positive control for PCR.

ER is present four times on the mycolactone plasmid. Although there is no evidence concerning plasmid copy number, most large plasmids are present in only 1 or 2 copies per cell making the copy number for the ER target 4–8 [3]. Because we initially assumed there was a clear correlation between copy number and PCR sensitivity, we were concerned that the lower copy number of the ER domain, with respect to IS*2404,* might influence the sensitivity of the method. Thus the relative sensitivity of ER and IS*2404* PCR was evaluated using 10-fold dilutions of *M. ulcerans* culture. As few as 10^−1^ CFU of *M. ulcerans* could be detected using either method (Figure 2). These results suggested that ER PCR was adequately sensitive for detection of *M. ulcerans* in environmental samples where few copies of *M. ulcerans* might be present.

### ER and VNTR primer sets are sensitive at low concentrations of *M. ulcerans* DNA

Sensitivity of the primer sets targeting VNTR loci and ER for environmental samples was also determined by spiking samples of belostomatids with serial dilutions of *M. ulcerans* DNA and performing ER and VNTR PCR. Results from this study show that ER and VNTR DNA could be detected at predicted concentrations as low as 0.1 CFU (Figure S2).

### ER PCR based evidence of *M. ulcerans* in environmental samples from endemic and non-endemic villages in Ghana

**Figure 3:**
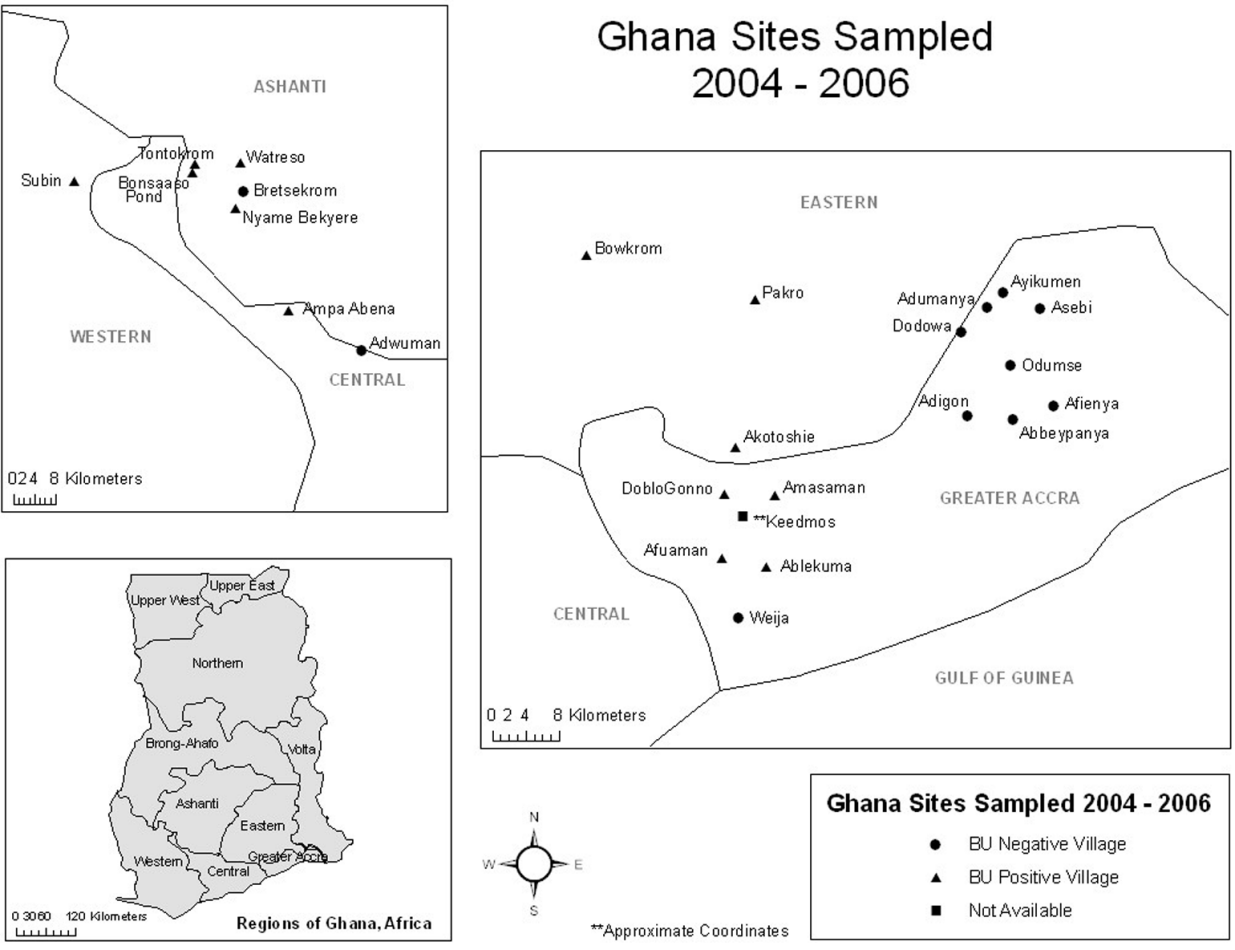
Sites sampled 2004–2006. Endemicity is based on human incidence of disease defined at the community level from data obtained from the Ghana Ministry of Health. **Location is approximate and endemicity is based on district level disease incidence data (GPS coordinates not available).

During 2004–2006 1,068 invertebrate and vertebrate samples were collected from 14 endemic and 12 non-endemic sites with a focus on the Ashanti and Greater Accra regions of Ghana (Figure 3). Samples included material collected within 1 m^2^ quadrats (N = 3) as well as those obtained by sweep sampling through vegetation. Identical sampling methods were used for all sites. Endemic sites yielded more samples than non-endemic sties. Of the 1,068 samples obtained, 572 (54%) were obtained from endemic sites whereas 496 (46%) were from non-endemic sampling sites. *M. ulcerans* DNA was detected in only 7% (78/1,068) of the total samples (Table 4) using ER PCR. From the 78 ER positive samples, 42 (54%) were from aquatic environments endemic for Buruli ulcer; whereas 36 (46%) samples were from non-endemic sites. The largest number of ER positive invertebrate samples was collected from Afuaman where 18 invertebrate pooled or individual samples were found to be positive. Six sites yielded only one ER positive pooled or individual sample. These included three endemic sites (Tontokrom, Bowkrom, and Amasaman) and three non-endemic sites (Bretsekrom, Dodowa, and Keedmos). Eight sites yielded zero ER positive invertebrate or vertebrate samples (five endemic and three non-endemic). The remaining eleven sites (six non-endemic and five endemic) had a range of 2 to 10 PCR positive pooled or individual invertebrate samples. All ER PCR positive results were confirmed by DNA sequencing.

**Table 4:** Detection of *M. ulcerans* and MPM in environmental samples using ER and VNTR PCR.

Samples	ER positive (%)	*M. ulcerans* VNTR profile (%)	MPM VNTR profile (%)
Invertebrate/vertebrates	78/1068 (7)	12/67 (18)	3/67 (4)
Water filtrate	97/260 (37)	8/82 (10)	4/82 (5)
Biofilm	37/47 (79)	17/37 (46)	8/37 (22)
Soil	3/100 (3)	0/3 (0)	0/3 (0)

ER positive DNA was detected in a broad spectrum of vertebrates and invertebrates representing 30 of the 89 taxa identified. Many taxa, such as Crambidae (moth) larvae and Araneae were found repeatedly positive at specific sites during the 2 year sampling period. Two pools of Crambidae larvae were found positive from Subin; one collected 2005 and the other collected 2006. Araneae have been found positive from sampling of Amasaman 2004, 2005, and 2006. Although some taxa, such as Belostomatidae and Naucoridae have been found IS*2404* positive by others [12],[13],[14] most ER PCR positive taxa reported in this study have not previously been identified as potential sources of *M. ulcerans*. *M. ulcerans* positive taxa represented a wide variety of functional invertebrate feeding groups and life stages (Table 2) [30]. Although most of the positive taxa represented predators, positive results were obtained from collector-gatherers such as those from the family Elmidae (beetle) and scrapers such as those from the family Baetidae (mayfly). A complete description of the demography and identification of positive taxa per site are presented in a separate paper (in preparation).

Previous reporting of *M. ulcerans* in Belostomatidae and Naucoridae led us to selectively collect additional samples from these taxa. Seventy-one additional belostomatids and twenty additional naucorids were obtained through selective collection. Of those, 3/71 (4%) belostomatids and 7/20 (35%) naucorids were found to contain ER positive DNA.

Although these results suggest that *M. ulcerans* DNA is widely distributed in invertebrates, the majority of taxa identified (59/89) were repeatedly negative for *M. ulcerans* DNA (Table S1). In some cases where a taxon was represented by a single sample, such as with Calonoida (copepod), little can be said about the absence of *M. ulcerans.* In other cases such as with Coenagrinidae (damselfly larvae) and Pleidae (backswimmer), over 100 individuals were sampled. The absence of ER PCR positive results from these taxa is more meaningful.

Out of 260 samples of water filtrate tested (130 from non-endemic and 130 from endemic sampling sites), 97 (36%) were ER PCR positive. Sixty of the 97 ER positive filtrate samples (61%) were from areas non-endemic for Buruli ulcer, while 37 (38%) of the ER positive filtrate samples were from endemic areas. PCR was also conducted on 100 soil samples; 50 of which were from endemic sites and 50 from non-endemic sites. *M. ulcerans* DNA was detected in 3% (3/100) of the soil samples (Table 4). Each of these three samples was collected from the floor of the water body. Two of the three ER PCR positive soil samples were from an area endemic for Buruli ulcer (Nyame-Bekyere and Subin) while the third was from an area non-endemic for Buruli Ulcer (Abbeypanya).

### VNTR analysis reveals heterogeneity with *M. ulcerans* and distinguishes *M. ulcerans* from other MPM

Although ER PCR is a reasonable preliminary test for the identification of *M. ulcerans*, the discovery of other mycolactone producing mycobacteria (MPM) in fish and frogs revealed that mycolactone genes are not *M. ulcerans* specific [26],[31]. In order to distinguish between *M. ulcerans* and other MPM, a VNTR-based method was developed based on published VNTR sequence [23],[24],[25]. For this analysis, a panel of 6 Ghanaian *M. ulcerans* isolates obtained from patients in the same regions where the environmental samples were collected was compared to a panel of MPM species. Primers targeting VNTR loci 4, 8, 14, 15, 18, and MIRU 9 did not distinguish between Ghanaian isolates of *M. ulcerans* and other MPM, although several of these loci had been previously used to discriminate between Beninese *M. ulcerans* and other MPM [24],[25].

Although some studies have found only 1 biovar of *M. ulcerans* in West Africa suggesting very little heterogeneity among *M. ulcerans* isolates within Africa [24],[25] one paper, which investigated a large group of *M. ulcerans* isolates from Ghana identified three different biovars [23]. In this paper, VNTR analysis of 6 *M. ulcerans* isolates from the Greater Accra, Central and Ashanti regions revealed three *M. ulcerans* VNTR profiles, A, B, and C based on MIRU 1, locus 6 and STI (Table 5). Profile A strains contained one copy of MIRU 1, one copy of locus 6, and one copy of ST1 (1,1,1). Profile B strains had three copies of MIRU 1, one copy of locus 6, and one copy of ST1 (3,1,1) and profile C consisted of a single isolate with three copies of MIRU 1, one copy of locus 6, and two copies of ST1 (3,1,2). Two of these VNTR profiles, B and C, were previously identified by Hilty *et al* [23] whereas profile A, characterized by a single copy of MIRU 1 and one copy of ST1 represented a new profile.

**Table 5:** VNTR profiles of *M. ulcerans* and other MPM isolates based upon numbers of repeats found at different loci (MIRU1, locus 6, ST1, and Locus 19 when applicable).

	VNTR Profile	MIRU1	Locus 6	ST1	Locus 19
***M. ulcerans*** (Human Ghanaian isolates)					
1054	B	3	1	1	
1055	B	3	1	1	
1057	B	3	1	1	
1063	B	3	1	1	
Agy99	C	3	1	2	
1059	A	1	1	1	
***M. marinum*** (Marine fish isolates)					
DL150991	D	1	4	2	2
DL240490	D	1	4	2	2
DL045	D	1	4	2	2
DL180892	D	1	4	2	2
SA200695	D	1	4	2	2
SR030597	D	1	4	2	2
CF030494	D	1	4	2	2
2790995	D	1	4	2	2
DL300/04 (Italy from Concrete)	E	1	2	1	2
CC240299 (Freshwater fish isolates)	E	1	2	1	2
BB170200 (Freshwater fish isolates)	E	1	2	1	2
***M. pseudoshottsii*** (freshwater fish)					
L15	D	1	4	2	2
L58	D	1	4	2	2
***M. liflandii*** (African, US imported frogs)					
Xt128f	F	1	2	2	1
Xl5	F	1	2	2	1
1138	F	1	2	2	1

These VNTR loci also distinguished *M. ulcerans* from other MPM (Table 5). Finally, the addition of locus 19 made it possible to distinguished *M. liflandii*, a newly discovered frog pathogen, from mycolactone producing fish pathogens *M. marinum* and *M. pseudoshottsii* (Table 5).

Two separate VNTR profiles were identified among mycolactone producing *M. marinum* isolates and these were associated with different habitats (Table 5). Whereas fish from salt water had profile D, those from freshwater had profile E (Table 5). Profile D included one copy of MIRU1, four copies of locus 6, two copies of ST1, and two copies of locus 19 (1,4,2,2), and profile E had one copy of MIRU1, two copies of locus 6, one copy of ST1, and two copies of locus 19 (1,2,1,2). Despite the great geographical distance between the Red and Mediterranean Seas and the Chesapeake Bay, MPM *M. marinum* isolated from sea bass (*Siganus nivulatus*) and *M. pseudoshottsii* isolated from striped bass (*Morone saxatilis*) shared identical 1,4,2,2 VNTR profiles. VNTR analysis revealed a single VNTR profile for *M. liflandii* (1,2,2,1). These results showed that VNTR could be used to differentiate MPM found in environmental samples in Ghana.

### *M. ulcerans* and other MPM are present in both Buruli ulcer endemic and non-endemic sites

To discriminate between *M. ulcerans* and other MPM, 78 ER-PCR positive samples collected from standardized sampling and 10 ER positive belostomatids and naucorids (3 belostomatids and 7 naucorids) that were selectively collected were tested for the presence and copy number of MIRU1, locus 6, ST1, and, if applicable, locus 19. Of these samples, VNTR profiles were obtained from 67 invertebrate/vertebrate samples (Table 6). The remaining 31 samples could not be VNTR typed presumably due to insufficient material. VNTR profiling showed that only 12 of these 67 samples (18%) had a VNTR profile which matched *M. ulcerans* (Table 6). Seven of these were collected from aquatic environments endemic for Buruli ulcer, and five of these were from non-endemic water bodies. *M. ulcerans* Profile A was identified in 9 different invertebrate species, whereas *M. ulcerans* profile C, found in the genome sequence strain *Agy99* was detected in specimens of a Nepidae (Order Hemiptera), a Belostomatidae and an unidentified spider. VNTR MPM profile D was found in three samples, including a tadpole (Anura) and two predacious aquatic insects (Coleoptera: Families Hydrophilidae and Dytiscidae, Table 4). *M. ulcerans* VNTR profile A and MPM profile D was obtained from different samples of Dytiscidae, Anura and Hydrophilidae. Both Anura and Hydrophilidae samples were collected from the same endemic site. The Dytiscidae samples were collected from two different endemic sites. *M. ulcerans* profiles A and C were identified in two separate Belostomatidae samples collected from separate sites, one endemic and one non-endemic. These results suggest that *M. ulcerans* and other MPM occupy the same water body.

**Table 6:** VNTR analysis of ER positive samples.

Order	Family	Description	Site Endemicity	VNTR profile	VNTR Profile Designation
Hemiptera	Nepidae	Predacious aquatic insect	N	MU 3,1,2	C
Araneae		Spider-predacious	N	MU 3,1,2	C
Hemiptera	Belostomatidae	Predacious aquatic insect	N	MU 3,1,2	C
Hemiptera	Belostomatidae	Predacious aquatic insect	E	MU 1,1,1	A
Anura		Tadpole	E	MU 1,1,1	A
Lepidoptera	Crambidae	Moth larvae	E	MU 1,1,1	A
Odonata	Libellulidae	Predacious aquatic insect	N	MU 1,1,1	A
Coleoptera	Dytiscidae	Predacious aquatic insect	N	MU 1,1,1	A
Diptera	Culicidae	Mosquito pupae	E	MU 1,1,1	A
Hemiptera	Notonectidae	Predacious aquatic insect	E	MU 1,1,1	A
Odonata	Protoneuridae	Damselfly larvae	E	MU 1,1,1	A
Coleoptera	Hydrophilidae	Predacious aquatic insect	E	MU 1,1,1	A
Anura		Tadpole	E	MPM1,4,2,2	D
Coleoptera	Hydrophilidae	Predacious aquatic insect	E	MPM1,4,2,2	D
Coleoptera	Dytiscidae	Predacious aquatic insect	E	MPM1,4,2,2	D

Profiles A–C: *M. ulcerans* (MU); Profile D: MPM associated with fish N: non-endemic, E: endemic.

VNTR analysis of 82 ER PCR positive water filtrates yielded 8 *M. ulcerans* positive samples. One of these was profile B whereas the other 7 typed as profile A. Four of these samples were from non-endemic areas, while the remaining four samples were from endemic areas. Four of the 82 ER PCR positive water filtrates yielded MPM profile E. Two of these were from endemic regions whereas two were from non-endemic sites. The identity of all VNTR products was confirmed by sequence analysis. Representative gels illustrating VNTR profiles from various sample types are given in Figure S1. These data suggest that human endemicity data do not reliably predict the presence of *M. ulcerans* in Ghana.

### Physical evidence consistent with the presence of mycobacteria can be obtained by collection of biofilm communities on glass slides

**Figure 4:**
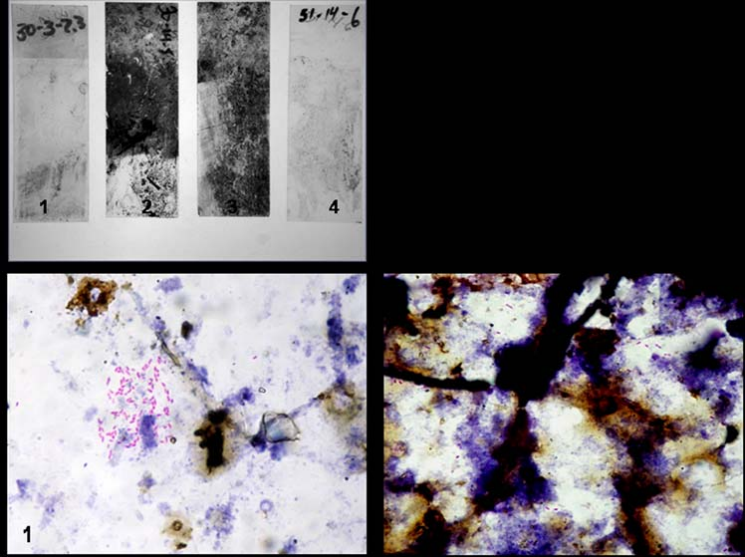
Collection of bacterial biofilms on glass slides from aquatic environments. (A) Slide 1, Adigon: 3 weeks, slide 2, Adigon: 14 weeks, slide 3, Amasaman: 3 weeks, slide 4, Amasaman: 14 wks. (B) 1, Acid-fast stain of bacilli found on a slide in which VNTR profiling matched *M. liflandii*. 2, Acid-fast stain of bacilli found on a slide in which VNTR profiling matched *M. ulcerans*. Acid-fast bacilli shown are representative of those found on most collected slides.

Ninety-six glass slides were submerged in water bodies associated with human use in the communities of Amasaman (endemic) and Adigon, (non-endemic). From these, 47 slides were collected at 21, 42 and 98 days. At 21 days, biofilm formation on slides collected from Adigon was sparse, but became progressively denser over the course of the experiment. In contrast, at Amasaman, the endemic site, biofilms were very dense by 21 days, but became less dense over the course of the study (Figure 4). Acid-fast bacilli were found on 45 of 47 slides (Figure 4). Microscopic analysis of the biofilm community showed the presence of diatoms and fungus as well as a mixed population of bacteria and considerable detritus. Acid-fast bacilli occurred in clusters or small groups, but were not associated with other flora present on the slide consistent with the ability of mycobacteria to adhere to glass [32].

### Adigon (non-endemic)

**Figure 5:**
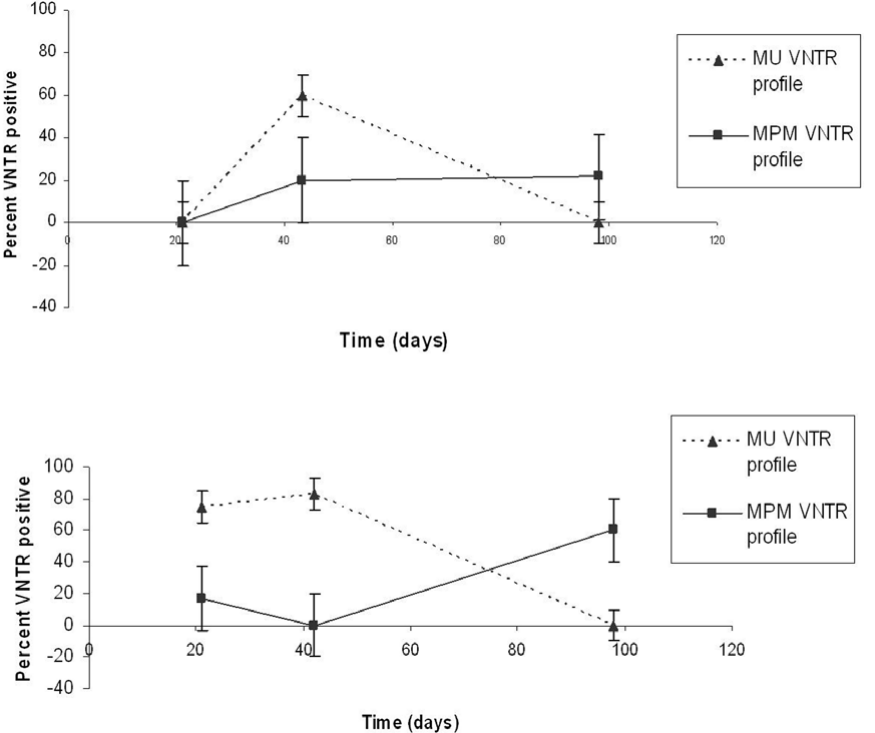
A timecourse of VNTR profiles of *M. ulcerans* or other mycolactone producing mycobacteria from biofilm slide samples collected from a Buruli ulcer endemic and non-endemic aquatic site. (Top) Percent of ER-positive biofilm slides with *M. ulcerans* or MPM VNTR profiles at 21d, 42, and 98d from A-Adigon (non-endemic). (Bottom) Percent ER-positive biofilm slides collected at 21d, 42d, and 98d from Amasaman (endemic).

Of the 47 biofilm slides analyzed, 37 were ER PCR positive (Table 4). VNTR profiles of 17 (46%) of these matched *M. ulcerans*, while 8 matched VNTR profiles of other MPM. VNTR analysis of slides collected from Adigon at 21 days was not conducted because all samples were ER negative (Figure 5). Three of five ER positive slides (60%) collected at 42 days from Adigon had *M. ulcerans* VNTR profile A, whereas one of the slides had a VNTR profile matching other MPM (profile D*). M. ulcerans* VNTR profiles were not found at Adigon at 98 days although VNTR patterns matching MPM were found on two slides. One of these corresponded to *M. liflandii* (profile F) while the other matched that of MPM associated with fish (profile E).

### Amasaman (endemic)

Nine of the twelve (75%) ER positive slides taken from Amasaman at 21 days had a *M. ulcerans* VNTR profile matching profile A, whereas a VNTR profile matching that of *M. liflandii* (profile F) was found on two slides. Five of six (83%) ER positive slides taken at 42 days from Amasaman had *M. ulcerans* profiles. *M. ulcerans* was not detected on the slides taken from Amasaman at 98 days although three slides (60%) produced VNTR signatures matching fish-associated MPMs (D and E). These results show the evolution of biofilm communities through time. The absence of *M. ulcerans* at 98 days is particularly interesting and could be explained by spontaneous detachment of the biofilm, or by grazing by tadpoles or invertebrates.

### *M. ulcerans* DNA is not detected in ER negative environmental samples

The analysis of VNTR data from environmental samples is complicated by many factors not present when analysis is performed on a pure bacterial colony. DNA extracted from insects, frogs, fish or filters contains DNA from a complex population of organisms. If VNTR profiling is a valid tool for detection of *M. ulcerans* in environmental samples, ER negative samples should also be negative for *M. ulcerans* by VNTR PCR. If however, specific VNTR sequences are present in a number of different organisms, or in bacteria which do not produce mycolactone, VNTR analysis of ER negative sites could yield a *M. ulcerans* or MPM profile. For example, if 1 repeat of MIRU1, locus 6 and ST1 were present in each of three different bacteria within a single environmental sample, this sample would produce a VNTR profile consistent with *M. ulcerans*. If this were the case, VNTR analysis of environmental samples would have little value in the identification of *M. ulcerans.* To address this possibility, VNTR analysis was performed on two sets of ER negative samples. The first set consisted of ER negative DNA from 35 samples representing a broad spectrum of samples collected at many different sites. The second set of samples was a complete sample set of 34 samples from a single ER negative site. Invertebrate, vertebrate, water filtrate, and soil samples were represented in each set. Though some of these samples produced bands for an individual locus, none of these samples produced a *M. ulcerans* VNTR profile.

### *Mycobacterium ulcerans* and MPM are widely distributed within endemic and non-endemic sites in the Ashanti and Greater Accra regions

Twenty-six sites were sampled from 2004–2006 (Table 7). Fourteen were endemic and twelve were non-endemic. These sites represented water bodies from south-central regions in Ghana with a focus on the Greater Accra and the Ashanti regions. All samples from seven sites were ER negative suggesting the absence of any MPM including *M. ulcerans*. Six sites had samples with DNA insufficient for VNTR analysis. VNTR profiling was performed on the remaining thirteen sites, seven endemic and six non-endemic sites. *M. ulcerans* profile A was found in 6 of the endemic sites. Three endemic sites had only one VNTR profile: Ampa Abena and Nyame-Bekyere had *M. ulcerans* profile A, and Subin had MPM profile E. Two or more VNTR profiles were found within the same water body at four of the endemic sites. Bonsaaso was found to contain *M. ulcerans* VNTR profiles A, B and C. Along with *M. ulcerans* profile A, Bowkrom and Afuaman also had MPM profiles E and D, respectively. Amasaman was found to contain two *M. ulcerans* VNTR profiles (A and B), one of the MPM *M. marinum* VNTR profiles (profile D), and the profile corresponding to *M. liflandii* (profile F).

**Table 7:** ER and VNTR results from all samples obtained from endemic and non-endemic sites sampled.

ENDEMIC			
Site/Community Name	District	ER pos	VNTR Profile
Pakro	Akwapim S	+	NA
Bowkrom	Akwapim S	+	A,E
Amasaman	Ga	+	A,B,D, F
Afuaman	Ga	+	A,D
Nyame Bekyere	Amansie W	+	A
Tontokrom	Amansie W	+	NA
Bonsaaso River	Amansie W	-	ND
Bonsaaso Pond	Amansie W	+	A,B,C
Ampa Abena	Denkyira	+	A
Subin	Denkyira	+	E
DobloGonno	Ga	-	ND
Ablekuma	Ga	-	ND
Watreso	Amansie W	-	ND
Akotoshie	Ga	-	ND

NONENDEMIC			
Site/Community Name	District	ER Pos	VNTR Profile
Dodowa	Dangme W	+	NA
Ayikumen	Dangme W	-	ND
Adumanya	Dangme W	-	ND
Asebi	Dangme W	+	A
Keedmos*	Ga	+	NA
Odumse	Dangme W	+	A
Abbeypanya	Dangme W	+	C
Afienya	Dangme W	+	E
Weija	Ga	+	NA
Adwuman	Denkyira	+	A
Bretsekrom	Amansie W	+	NA
Adigon	Ga	+	A,D,E,F

ND: VNTR not performed. NA: Insufficient DNA for VNTR profiling. Profiles A–C: *M. ulcerans;* Profiles D–F: other mycolactone-producing mycobacteria.

*: Endemicity based upon district rather than community level incidence of human disease.

Samples from six non-endemic sites produced VNTR profiles. However, there was less diversity of VNTR profiles from the non-endemic sites than endemic sites. Four of these sites were represented by one *M. ulcerans* profile (either profile A or C), and one of the sites, Afienya, had only a MPM *M. marinum* VNTR profile (profile E). Adigon was the only non-endemic site which yielded multiple VNTR profiles. VNTR profiles of *M. ulcerans* (profile A), MPM *M. marinum* (profiles D and E), and *M. liflandii* (profile F) were all obtained from biofilm samples collected in Adigon.

VNTR profiles representing *M. ulcerans* and other MPMs were obtained from sites from both the Greater Accra and the Ashanti regions (Figure 3). *M. ulcerans* and MPM VNTR profiles were found within the same site more frequently in the Greater Accra region than in the Ashanti region. *M. ulcerans* VNTR profiles A, B and C (1,1,1, 3,1,1 and 3,1,2 respectively) were found in both the Greater Accra and the Ashanti regions. MPM *M. marinum* profile D was found only in the Greater Accra region, whereas profile E was found in both regions. Profile F (*M. liflandii)* was found in two sites of the Greater Accra region.

## Discussion

In this paper we present results from a large scale study of *M. ulcerans* in the environment. Although a number of studies have reported the presence of *M. ulcerans* in environmental samples from endemic regions [13],[16],[17],[33], this is the first study where standardized ecological methods were used to reduce sampling bias, and the first to include longitudinal data from both Buruli ulcer endemic and non-endemic sties. One of the mysteries of Buruli ulcer is the close proximity of endemic and non-endemic villages. For example, whereas the disease is rarely reported from the Ga East district of the Greater Accra region in Ghana, it is endemic in the Ga West district despite the fact that endemic and non-endemic villages may be separated by only a few kilometers (Figure 3). Since the climate, rainfall, plant populations and ethnic groups in Ga East and Ga West are similar it has been difficult to understand the differential occurrence of Buruli ulcer within these regions.

The most important finding from this study is that, whereas Buruli ulcer occurs within discrete geographic village foci within endemic regions, *M. ulcerans* is widely distributed in water bodies in both endemic and non-endemic villages in the Greater Accra and Ashanti regions. This is consistent with its position as an environmental pathogen. We have also been able to repeatedly detect the presence of *M. ulcerans* within some sites over a 27 month framework suggesting the long term survival and presence of *M. ulcerans* in specific locations. These results clearly show that the focal occurrence of Buruli ulcer within the Greater Accra and Ashanti regions cannot be explained by the presence or absence of *M. ulcerans* in the environment. Thus other factors such as demography and human behavior may be important in the epidemiology of Buruli ulcer in these regions.

In contrast, there are large geographic areas in West Africa such as the Volta region of Ghana, or drier Northern parts of Ghana, Benin and Togo, where Buruli ulcer has never been reported. It has been assumed that the absence of Buruli ulcer from these regions is based on environmental constraints which restrict the growth of *M. ulcerans* or potential reservoir species. Results from an on-going project in the Volta region confirm this hypothesis in that we have failed to reveal a single *M. ulcerans* positive sample out of hundreds of invertebrate, water filtrate or macrophyte samples from 20 sites sampled. The absence of Buruli ulcer in Volta is explained by the absence of *M. ulcerans* (work in progress).

In a clinical setting the use of a single PCR target for detection of a pathogen is widely accepted. However, the use of a single PCR target for identification of bacteria in an environmental sample is rarely adequate. In Ghana, analysis of many IS*2404* positive samples revealed the presence of mycolactone producing mycobacterial species (MPMs) other than *M. ulcerans* as had been predicted [29]. In contrast, in Australia IS*2404* PCR appears to be specific for *M. ulcerans* because of the absence of other MPMs [33]. Here we provide the first evidence for the presence of MPMs in West Africa and show that MPMs and *M. ulcerans* share aquatic environments. The pathogenic potential of MPM for humans is unknown, although the lower growth temperature of some of these species makes them unlikely human pathogens [26]. The fact that the strain complexity of MPMs and *M. ulcerans* is greater in endemic areas and greatest within the Greater Accra region is an intriguing finding which needs further investigation.

The use of geographic-specific VNTR profiles in following chains of transmission is extremely important since the heterogeneity of *M. ulcerans* isolates appears to differ within different West African countries [23],[24],[25]. For example, data based primarily on patient isolates from Benin led to the conclusion that there was a single West African *M. ulcerans* clone. However, several biovars have been identified in Ghana [23]. Our results agree with those of Hilty *et al* [23] in showing the presence of that at least 3 different VNTR profiles in Ghana. Thus it is important when discriminating between *M. ulcerans* and other MPM that a geographically representative set of patient isolates is used.

Our initial concerns regarding the effect of low target copy number on the sensitivity of PCR methods reflected our naiveté regarding PCR theory. We had not considered that the efficiency of the PCR reaction depends on many factors including the efficiency of primer binding, the length of the product and local DNA conformation or that because the reaction is exponential, the first few targets bound may rapidly become the major products. Experimental results confirm this theory since others have found no difference between the use of IS*2404* PCR and that of 16sRNA PCR for detection of *M. ulcerans* in human samples despite the enormous difference in copy number [34] and results from VNTR analysis of clinical isolates show gel bands with an intensity never reported for *IS2404* PCR [23] . It is possible that the fact that VNTR sequences are non-coding segments of DNA may make them more accessible to primer binding.

Our studies confirm the presence of *M. ulcerans* in predacious aquatic insects including Belostomatidae and Naucoridae families reported by Portaels *et al* [15] and extend these findings by showing that VNTR profiles from these insects match those of human isolates of *M. ulcerans*. Belostomatids were common in many sites sampled throughout the year. However, even where large numbers of Belostomatidae were collected the rate of *M. ulcerans* infection was very low. In Ghana, despite repeated seasonal sampling the numbers of naucorids found were very low (paper in preparation). Evidence for the role of naucorids as potential *M. ulcerans* vectors comes from studies in Cote d'Ivoire [16]. Insect population studies are needed to confirm whether naucorids are more abundant in Cote d'Ivoire than in Ghana.

Our results show that *M. ulcerans* is widely distributed within invertebrate communities in aquatic environments. However, none of the *M. ulcerans*-positive, predacious invertebrates are hematophagous; thus the frequency with which humans are bitten would be expected to be quite low [35]. Although potential trophic relationships exist between several taxa studied (belostomatids, for example, feed on many other invertebrates and vertebrates and also cannibalize each other), it will take considerably more work to elucidate chains of transmission within the environment. Results presented here are based on determining the presence or absence of *M. ulcerans* in an environmental sample. Further studies need to be conducted using quantitative PCR methods to determine which species are most heavily infected and thus are more likely to serve as vectors.

Although it has been reported that snails and fish may harbor *M. ulcerans* [17] our results suggest the possibility that many of the IS*2404* positive mycobacteria detected are MPM other than *M. ulcerans*. In our studies *M. ulcerans* was never detected in fish or snails, although other MPM were identified in later studies. The most consistently *M. ulcerans*-positive samples detected were filtered water and biofilms on glass slides. This suggests that exposure of open lesions to infected water cannot be ruled out as a potential source of infection.

A general problem regarding detection of *M. ulcerans* in environmental samples is that evidence has come almost solely from detection of *M. ulcerans* DNA and under-estimation of *M. ulcerans* could result due to the presence of PCR inhibitors. Our results suggest that current methods are effective in eliminating PCR inhibitors since dilution of samples did not result in the detection of many additional ER positive samples and none of those detected through dilution could be confirmed by sequencing. Despite the broad spectrum of samples we did not find evidence for inhibitors in any particular taxa or sample type tested. Nonetheless, the possibility exists that the number of positive *M. ulcerans* positive samples may be underestimated.

The use of slide biofilms for trapping mycobacteria in the environment has proven particularly useful since it provides preliminary physical evidence for the presence of mycobacteria (AFB staining) along with molecular evidence, and facilitates longitudinal studies. The numbers of slides used and placement of PVC pipes are crucial because of the inevitable loss of slides through changes in water level, or disturbance by animals or humans. There was a decrease of DNA samples from slides giving a VNTR profile matching *M. ulcerans* between 42 and 98 days in the two water bodies (Figure 5). There was, however, an increase in DNA samples from slides producing a VNTR profile matching other MPM s. These data suggest bacterial community dynamics between *M. ulcerans* and other MPMs.

In summary, we have developed new methods for mapping the distribution of *M. ulcerans* in aquatic environments and applied these in the Greater Accra and Ashanti regions of Ghana. This work is part of a much larger five year project in which data from water chemistry, LandSat satellite imaging of land cover, and macrophyte and aquatic invertebrate population structure will be used to define the broad ecology of *M. ulcerans*. The presence of *M. ulcerans* in both endemic and non-endemic villages within endemic regions suggests that studies of human ecology will be necessary to unravel the mysteries surrounding the transmission of *M. ulcerans* to humans. Our goal in this work is to define the *M. ulcerans* environment in order to develop programs for preventing human exposure. The findings presented here show the possibility of tracing transmission of *M. ulcerans* from the environment to humans. This work represents a small step towards solving the mysteries surrounding human infection.

## Supporting Information

Figure S1 ER PCR and VNTR profiling of representative samples collected 2004–2006. A. ER PCR of various sample types. Lanes are labeled 1: 1KB ladder; 2: Water blank DNA extraction; 3: Water blank PCR; 4: Water filtrate (Bonsaaso Pond); 5: Biofilm (Amasaman 21 days); 6: Dytiscidae (Afuaman); 7: Protoneuridae (Ampa Abena); 8: Baetidae (Bonsaaso River); 9: *M. ulcerans* Agy99. 10: *M. marinum* 1218; 11–12: empty. B–E. PCR targeting VNTR loci: (B) MIRU 1, (C) locus 6, (D) ST1, and (E) locus 19. Lanes for B–E are labeled 1: 1KB ladder; 2: water blank DNA extraction; 3: water blank PCR; 4: Water filtrate (Bonsaaso Pond); 5: Biofilm (Amasaman 21 days); 6: Dytiscidae (Afuaman); 7: Protoneuridae (Ampa Abena); 8: Baetidae (Bonsaaso River); 9: *M. ulcerans* 1063; 10: *M. ulcerans* 1059; 11: *M. marinum* DL240490; 12: *M. liflandii* 1138.
(0.05 MB JPG)

Figure S2 ER PCR and VNTR profiling of belostomatid samples spiked with dilutions of *M. ulcerans*. A. ER PCR of belostomatid samples spiked with serial dilutions of *M. ulcerans* 1615. B–E. VNTR analysis of MIRU 1 (B), Locus 6 (C), ST1 (D), and Locus 19 (E) of belostomatid samples spiked with serial dilutions of *M. ulcerans* 1615. All lanes are labeled 1: 1KB ladder; 2: water blank DNA extraction; 3: water blank PCR; 4: belostomatid with no *M. ulcerans* added; 5: belostomatid with predicted 10^5^ CFU M. *ulcerans* 1615; 6: belostomatid with predicted 10^4^ CFU *M. ulcerans* 1615; 7: belostomatid with predicted 10^3^ CFU *M. ulcerans* 1615; 8: belostomatid with predicted 10^2^ CFU *M. ulcerans* 1615; 9: belostomatid with predicted 10 CFU *M. ulcerans* 1615; 10: belostomatid with predicted 1 CFU *M. ulcerans* 1615; 11: belostomatid with predicted 0.1 CFU *M. ulcerans* 1615; 12: belostomatid with predicted .01 CFU *M. ulcerans* 1615; 13: *M. ulcerans* 1615.
(0.05 MB JPG)

Table S1 Pooled or individual organisms sampled in Ghana 2004–2006 from a particular taxon that were found to be ER PCR negative. ^1^Samples in total quantities above five were pooled in sets of 3–15. Denominator represents total number of pooled or individual samples collected from the specific taxon.
(0.08 MB DOC)
