# Supplementary figures and images for: Distribution of Mycobacterium ulcerans in Buruli Ulcer Endemic and Non-Endemic Aquatic Sites in Ghana
Source: PLoS Negl Trop Dis. 2008 Mar 26;2(3):e205. doi: 10.1371/journal.pntd.0000205 (PMC2268743; doi:10.1371/journal.pntd.0000205)

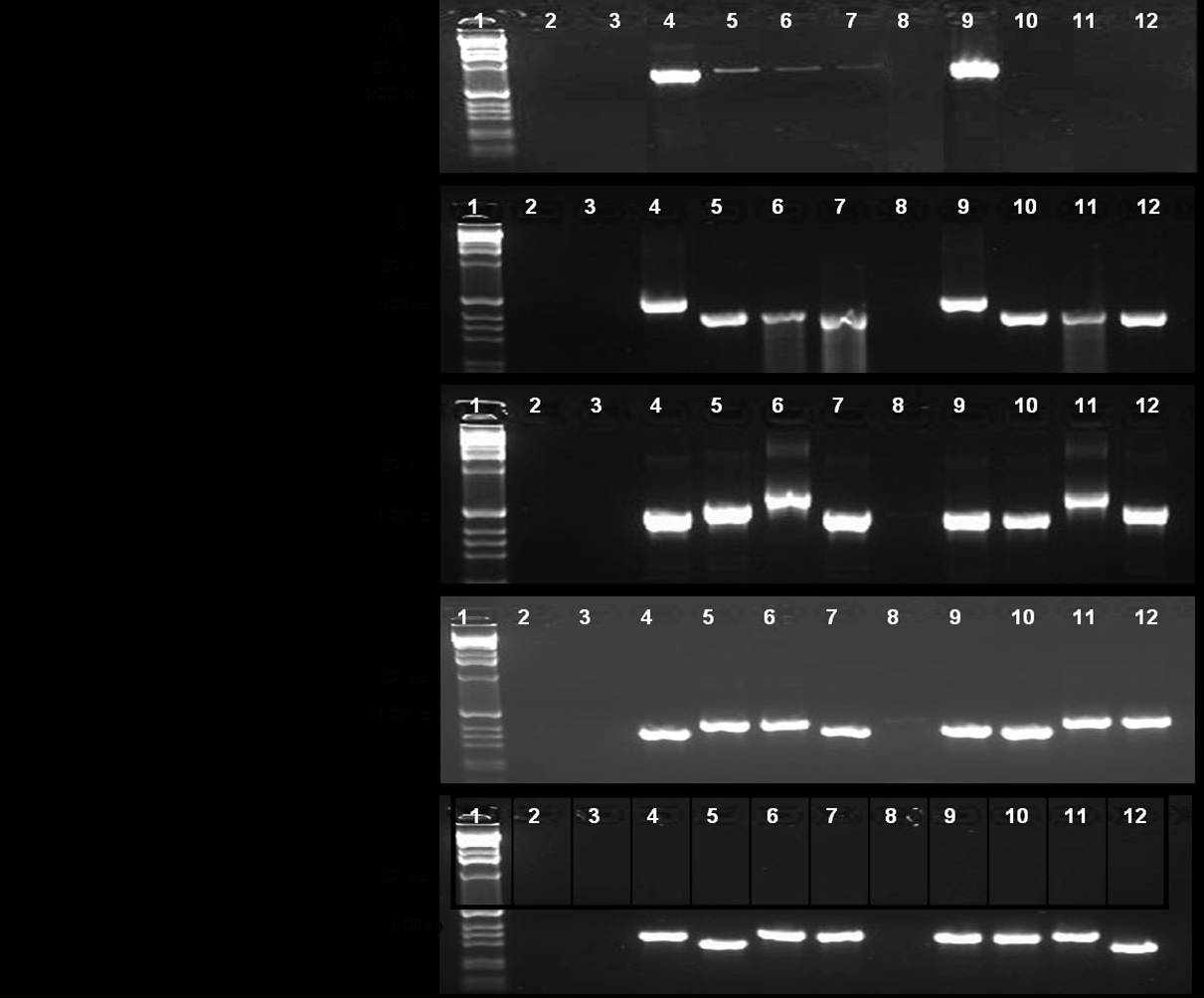

Supplement: Figure S1 — ER PCR and VNTR profiling of representative samples collected 2004–2006. A. ER PCR of various sample types. Lanes are labeled 1: 1KB ladder; 2: Water blank DNA extraction; 3: Water blank PCR; 4: Water filtrate (Bonsaaso Pond); 5: Biofilm (Amasaman 21 days); 6: Dytiscidae (Afuaman); 7: Protoneuridae (Ampa Abena); 8: Baetidae (Bonsaaso River); 9: M. ulcerans Agy99. 10: M. marinum 1218; 11–12: empty. B–E. PCR targeting VNTR loci: (B) MIRU 1, (C) locus 6, (D) ST1, and (E) locus 19. Lanes for B–E are labeled 1: 1KB ladder; 2: water blank DNA extraction; 3: water blank PCR; 4: Water filtrate (Bonsaaso Pond); 5: Biofilm (Amasaman 21 days); 6: Dytiscidae (Afuaman); 7: Protoneuridae (Ampa Abena); 8: Baetidae (Bonsaaso River); 9: M. ulcerans 1063; 10: M. ulcerans 1059; 11: M. marinum DL240490; 12: M. liflandii 1138. (0.05 MB JPG) [file pntd.0000205.s001.jpg]

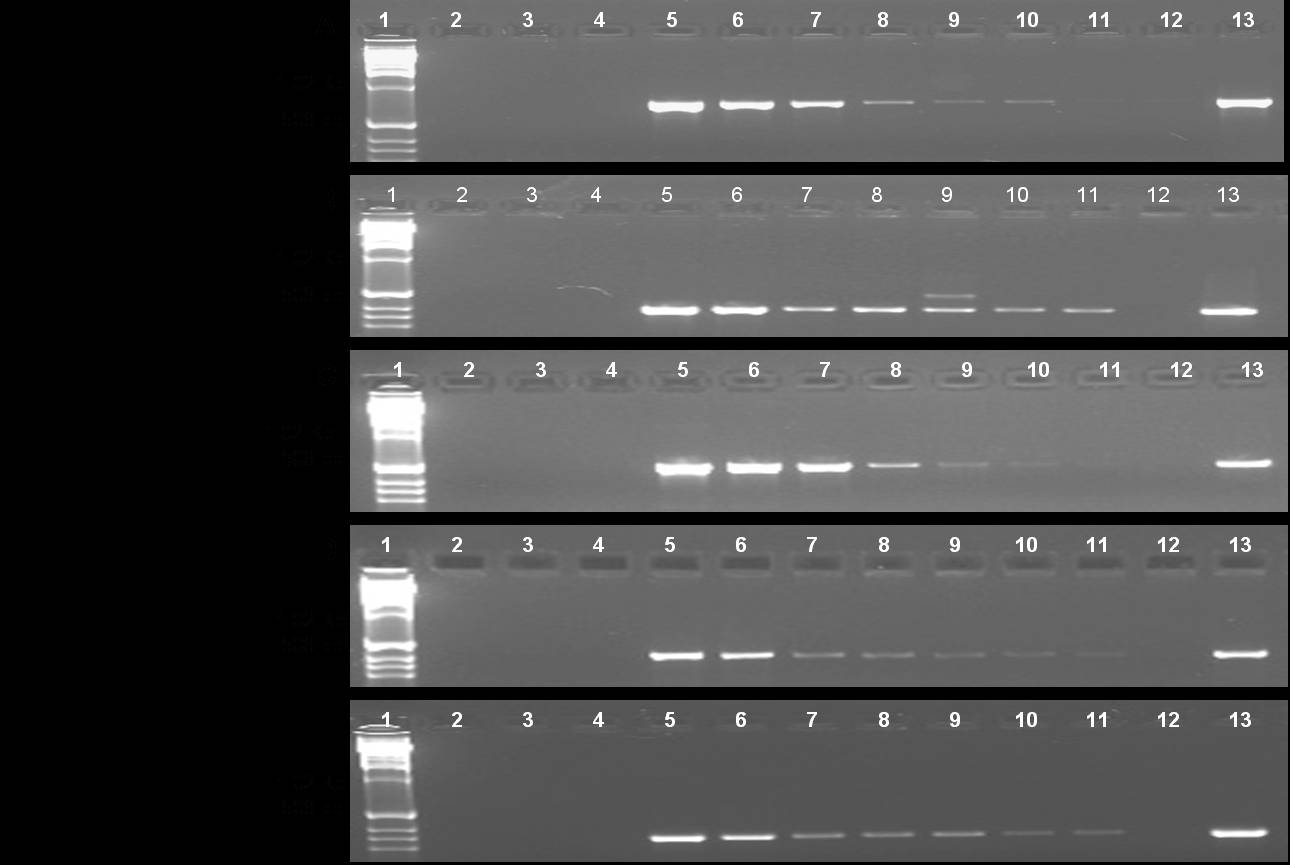

Supplement: Figure S2 — ER PCR and VNTR profiling of belostomatid samples spiked with dilutions of M. ulcerans. A. ER PCR of belostomatid samples spiked with serial dilutions of M. ulcerans 1615. B–E. VNTR analysis of MIRU 1 (B), Locus 6 (C), ST1 (D), and Locus 19 (E) of belostomatid samples spiked with serial dilutions of M. ulcerans 1615. All lanes are labeled 1: 1KB ladder; 2: water blank DNA extraction; 3: water blank PCR; 4: belostomatid with no M. ulcerans added; 5: belostomatid with predicted 105 CFU M. ulcerans 1615; 6: belostomatid with predicted 104 CFU M. ulcerans 1615; 7: belostomatid with predicted 103 CFU M. ulcerans 1615; 8: belostomatid with predicted 102 CFU M. ulcerans 1615; 9: belostomatid with predicted 10 CFU M. ulcerans 1615; 10: belostomatid with predicted 1 CFU M. ulcerans 1615; 11: belostomatid with predicted 0.1 CFU M. ulcerans 1615; 12: belostomatid with predicted .01 CFU M. ulcerans 1615; 13: M. ulcerans 1615. (0.05 MB JPG) [file pntd.0000205.s002.jpg]
