# Supplementary material for: Distribution of Mycobacterium ulcerans in Buruli Ulcer Endemic and Non-Endemic Aquatic Sites in Ghana
Source: PLoS Negl Trop Dis. 2008 Mar 26;2(3):e205. doi: 10.1371/journal.pntd.0000205 (PMC2268743; doi:10.1371/journal.pntd.0000205)
Supplement: Table S1 — Pooled or individual organisms sampled in Ghana 2004–2006 from a particular taxon that were found to be ER PCR negative. 1Samples in total quantities above five were pooled in sets of 3–15. Denominator represents total number of pooled or individual samples collected from the specific taxon. (0.08 MB DOC) [file pntd.0000205.s003.doc]

| **Order** | **Family** | **ER PCR^1^** |
| --- | --- | --- |
| Cladocera |  | 0/11 |
| Coleoptera | Lampyridae | 0/5 |
| Coleoptera | Gyrinidae | 0/2 |
| Coleoptera | Psephenidae | 0/1 |
| Coleoptera | Curculionidae | 0/1 |
| Coleoptera | Spercheidae | 0/3 |
| Coleoptera | Hygrobiidae | 0/1 |
| Collembola | Isotomidae | 0/2 |
| Collembola | Entomobryidae | 0/4 |
| Copepoda |  | 0/13 |
| Coleoptera | Unknown | 0/7 |
| Decapoda | Atyidae | 0/16 |
| Hemiptera | Saldidae | 0/2 |
| Diptera | Chaoboridae | 0/7 |
| Diptera | Tabanidae | 0/3 |
| Diptera | Stratiomyidae | 0/7 |
| Diptera | Dolichopodidae | 0/1 |
| Diptera | Tipulidae | 0/6 |
| Diptera | Ephydridae | 0/1 |
| Ephemeroptera | Heptageniidae | 0/4 |
| Ephemeroptera | Leptophlebiidae | 0/3 |
| Gastropoda | Viviparidae | 0/3 |
| Hemiptera | Gerridae | 0/17 |
| Hemiptera | Veliidae | 0/14 |
| Hemiptera | Hydrometridae | 0/11 |
| Hemiptera | Mesoveliidae | 0/34 |
| Hemiptera | Corixidae | 0/15 |
| Hemiptera | Pleidae | 0/24 |
| Hemiptera | Hebridae | 0/2 |
| Hydrozoa |  | 0/2 |
| Mysidacea | Mysidae | 0/1 |
| Nematoda |  | 0/3 |
| Odonata | Corduliidae | 0/5 |
| Diptera | Ceratopogonidae | 0/28 |
| Odonata | Coenagrinidae | 0/19 |
| Odonata | Aeshnidae | 0/3 |
| Odonata | Macromiidae | 0/2 |
| Odonata | Gomphidae | 0/1 |
| Odonata | Unknown Anisoptera | 0/17 |
| Odonata | Calopterygidae | 0/1 |
| Trichoptera | Leptoceridae | 0/6 |
| Trichoptera | Hydropsychidae | 0/2 |
| Trichoptera | Helicopsychidae | 0/1 |
| Trichoptera | Polycentropodidae | 0/1 |
| Gastropoda | Ancylidae | 0/4 |
| Gastropoda | Thiaridae | 0/8 |
| Gastropoda | Pilidae | 0/5 |
| Crustacea | Conchostraca | 0/1 |
| Gastropoda | Pleuroceridae | 0/5 |
| Emphemeroptera | Polymitarcyidae (Povilla) | 0/4 |
| Bivalva | Unionidae | 0/2 |
| Gastropoda | Lymnaedae | 0/2 |
| Collembola | Sminthuridae | 0/1 |
| Gastropoda | Unknown | 0/9 |
| Terrestrial insects |  | 0/12 |
| Ephemeroptera | Unknown | 0/1 |
| Odonata | Unknown Zygoptera | 0/13 |
| Lepidoptera | Noctuidae | 0/1 |
| Diptera | Unknown | 0/2 |
